# Physicochemical Properties of Indoor and Outdoor Particulate Matter 2.5 in Selected Residential Areas near a Ferromanganese Smelter

**DOI:** 10.3390/ijerph18178900

**Published:** 2021-08-24

**Authors:** Setlamorago Jackson Mbazima, Masilu Daniel Masekameni, Gill Nelson

**Affiliations:** Occupational Health Division, School of Public Health, University of the Witwatersrand, Parktown, Johannesburg 2193, South Africa; daniel.masekameni@wits.ac.za (M.D.M.); gill.nelson@wits.ac.za (G.N.)

**Keywords:** Meyerton, source apportionment, mass concentration, diameter, elemental composition, SEM-EDS, ICP-MS

## Abstract

Particulate matter (PM) of different sizes and elemental composition is a leading contributor to indoor and outdoor air pollution in residential areas. We sought to investigate similarities between indoor and outdoor PM_2.5_ in three residential areas near a ferromanganese smelter in Meyerton to apportion the emission source(s). Indoor and outdoor PM_2.5_ samples were collected concurrently, using GilAir300 plus samplers, at a flow rate of 2.75 L/min. PM_2.5_ was collected on polycarbonate membrane filters housed in 37 mm cassettes coupled with PM_2.5_ cyclones. Scanning electron microscopy coupled with energy-dispersive spectroscopy was used to study the morphology, and inductively coupled plasma-mass spectroscopy was used to analyse the elemental composition of the PM_2.5_. Mean indoor and outdoor PM_2.5_ mass concentrations were 10.99 and 24.95 µg/m^3^, respectively. Mean outdoor mass concentration was 2.27-fold higher than the indoor concentration. Indoor samples consisted of irregular and agglomerated particles, ranging from 0.09 to 1.06 µm, whereas outdoor samples consisted of irregular and spherical particles, ranging from 0.10 to 0.70 µm. Indoor and outdoor PM_2.5_ were dominated by manganese, silicon, and iron, however, outdoor PM_2.5_ had the highest concentration of all elements. The ferromanganese smelter was identified as the potential main contributing source of PM_2.5_ of different physicochemical properties.

## 1. Introduction

Particulate matter (PM) with an aerodynamic diameter of 2.5 µm or less (PM_2.5_) has been identified as the leading contributor to indoor and outdoor air pollution [1,2]. Although PM_2.5_ can be released from natural sources, anthropogenic sources such as mining activities, coal-fired power stations, motor vehicles, and smelters have been identified as major sources of atmospheric PM_2.5_ in residential areas [3,4,5]. High-temperature combustion sources, such as ferromanganese (FeMn) smelters, have been associated with high emissions of airborne manganese (Mn)-bearing PM_2.5_ with different physicochemical properties [6,7]. Higher concentrations of airborne Mn-bearing PM_2.5_ have been reported in residential areas downwind of FeMn smelters relative to upwind areas [8,9].

A study by Menezes-Filho et al. [10] measured atmospheric Mn-bearing PM_2.5_ in residential areas 1.3 km from a FeMn smelter plant in Salvador, Brazil. The authors reported atmospheric Mn concentrations ranging from 0.011 to 0.439 µg/m^3^. Total suspended particles atmospheric Mn sampled from 2003 to 2013 in two Ohio towns near a ferrosilicomanganese smelter were between 0.11 and 0.39 µg/m^3^, and 0.17 and 1.5 µg/m^3^ [11]. In another study, daily atmospheric Mn concentrations were found to be 1279 and 2062 ng/m^3^ in two residential areas near a FeMn smelter in Spain, Cantabria [7]. Most studies have focused largely on PM_2.5_ because it is suggested to have a higher resident time in the atmosphere relative to PM_10_ [8,12]. Furthermore, PM_2.5_ can be transported over longer distances from the source and enter indoor environments through infiltration and filtration mechanisms [13,14]. In modern society, people spend 80–90% of their time indoors where they are likely to be exposed. Specifically, pregnant women, sick people, toddlers, and the elderly have a weaker immune system and spend most of their time indoors, increasing the risk of exposure.

Epidemiological studies have shown a correlation between exposure to airborne Mn-bearing PM_2.5_ and cognitive and motor impairments, in residential areas near FeMn smelters [15,16,17,18,19]. A recent study [20] conducted near a FeMn smelter in Meyerton, South Africa, the site of the present study, reported an association between mood and environmental exposure to low Mn air concentrations. However, like other previous epidemiological studies [11,21,22,23], the study by Racette et al. [20] used data from ambient monitoring stations located farther from the receptors as a proxy for exposure for a specific population. Therefore, the studies lacked reliable exposure assessment data in the near field of the receptors [24,25]. The new era of exposure assessment argues that PM measured at or near the source may have different chemical signatures than that measured at far-field monitoring stations [26,27,28].

In South Africa, most of the conducted studies on exposure to airborne Mn [29,30] were confined to occupational settings. Rodríguez-Agudelo et al. [31] argued that although occupational settings commonly have higher airborne Mn concentrations than residential areas, occupational exposures are periodic, usually between 8 and 12 h/day. Conversely, residential exposures are continuous, usually between 12 and 24 h/per day, and include susceptible groups such as children under the age of five, the elderly, and immunocompromised people [31,32,33]. Davourie et al. [34], added that exposure to airborne Mn-bearing particles in occupational settings has been sufficiently investigated, however, exposure in residential areas remains a significant concern.

To the best of our knowledge, no studies have investigated the physicochemical properties of indoor and outdoor airborne PM_2.5_ in residential areas near FeMn smelters in South Africa [35]. Subsequently, there are limited data on physicochemical properties of PM_2.5_ and exposure assessment data in residential areas near FeMn smelters in South Africa [36,37]. Knowledge of the physicochemical properties of PM_2.5_ is important in understanding its origin, formation, and transformation mechanisms, and the processes that can occur at its surfaces [38]. Once the main emission source(s) have been identified, effective control measures to protect public health and the environment can be implemented [39,40]. The results may also provide insight into the morphology and elemental composition of the PM_2.5_ to which the residents are potentially exposed. This is important, given that the health outcomes of exposure to airborne Mn-bearing PM_2.5_ depend on the size and elemental composition [41]. In this study, we aimed to (1) characterise indoor and outdoor PM_2.5_; (2) determine the relationship between indoor and outdoor PM_2.5_ mass concentrations; and (3) apportion the emission source(s) of PM_2.5_ in three residential areas near a FeMn smelter in Meyerton, South Africa.

## 2. Materials and Methods

### 2.1. Description of Study Area

**Figure 1:**
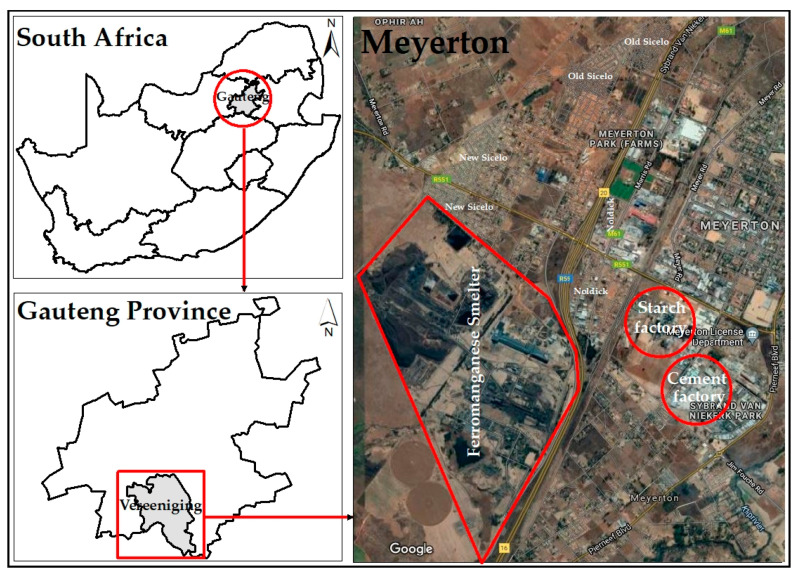
Geographical map of the study area (created using Google earth map (Mountain View, CA, USA) and Arcmap version 10.8 (Esri, Redlands, CA, USA)).

The study was conducted in the town of Meyerton with geographical positioning system coordinates 26.5854° S and 28.0069° E. Meyerton has an area of 180.24 km^2^ and had a population size of 55.283 people in the recent census [42]. The area has an average temperature of 17 °C, average rainfall of 34.4 mm, humidity 61%, and wind speeds of 6 km/h. Within Meyerton, there is a FeMn smelter that has been operating since 1959 and produces between 540 kilotons of FeMn, annually [43]. Old Sicelo, New Sicelo, and Noldick were the three residential areas selected for this study; all are located downwind of the FeMn smelter (Figure 1). Although the three residential areas are in the same region, they have different characteristics and are at different distances from the FeMn smelter. New Sicelo is closest to the FeMn smelter (~1.4 km), followed by Noldick (~1.5 km) and Old Sicelo (3.5 km).

In addition to the FeMn smelter, there are other stationery sources within the Meyerton area. A cement factory is located ~1.2 km from Noldick, 2.5 km from New Sicelo, and 2.8 km from Old Sicelo. There is also a coal-fired starch factory that is ~1.6 km from Noldick, ~3.3 km from New Sicelo, and ~3.6 km from Old Sicelo. Most dwellings at New and Old Sicelo are predominantly shacks, constructed from corrugated iron and boards; most of the dwellings in Noldick are constructed from cement blocks. The residential areas had been developed because people wanted to be closer to their workplaces to save money related to transport costs and to maximise their time [37]. Old and New Sicelo are located west of the R59, which is one of Gauteng Province’s busiest freeways that connects to other neighbouring provinces. The R551 and M61 roads that connect to other neighbouring residential areas pass through Noldick and New Sicelo.

### 2.2. Sampling of Indoor and Outdoor PM_2.5_

Polycarbonate membrane filters of 37 mm in diameter with a pore size of 0.08 µm were conditioned before and after sampling. Gravimetric weighing was undertaken under controlled laboratory conditions (at 21 °C temperature and 35% humidity), using an electronic microbalance scale (Sartorius, AG, Germany), model CPA225D, that has a minimum resolution and precision of 0.001 mg. Indoor and outdoor PM_2.5_ was sampled simultaneously, using two identical Gilian GilAir 300plus pumps (Sensidyne, St Petersburg, FL, USA). One pump was used to sample the outdoor PM_2.5_ while the other was used to sample indoor PM_2.5_ in the main activity room. Both pumps were placed indoors for security purposes and to protect them from harsh environmental conditions such as rain and direct sunlight, which can damage the pumps and affect their functionality. The pumps were connected to a Teflon tube that joined the pumps and a 37 mm cassette (SKC Inc., PA, USA) fitted with a polycarbonate membrane filter (PCTE) (Zefon, Ocala, FL, USA) [44].

The cassette was coupled with a 37 mm PM_2.5_ Gs-3 multiple-inlet cyclone (SKC Inc., PA, USA) that separated the coarse and fine particles, using centrifugal force. PCTE filters are suitable for microscopic analyses because they have a smooth surface area which makes it easier to detect single particles [45,46]. PCTE filters are also recommended for elemental analysis due to their low blank levels, inertness to gas adsorption, low impurities, low moisture absorption, high PM collection efficiency, and ability to withstand severe weather conditions [45,47].

Indoor and outdoor PM_2.5_ was sampled at a constant flow rate of 2.75 L/min and the particles were deposited onto the PCTE filters. Indoor and outdoor PM_2.5_ were sampled continuously for 24 h over seven days at 30 selected households, resulting in 60 samples (30 indoor and 30 outdoor samples). The sampled houses were randomly selected by drawing a grid on the study area map and two houses were selected from each grid. Sampling was conducted from September to November 2019, which was spring in South Africa.

Indoor PM_2.5_ samples were collected at a height of ~1.5 m, 1.2 m from the walls and openings, and 1 m from indoor sources such as cooking activities [48]. Indoor samples were collected in the sitting rooms; where impossible (e.g., one-room houses) indoor samples were collected in the middle of the room. There were no restrictions on the activities undertaken during the sampling, therefore, participants carried out their normal activities. For outdoor samples, the 1.5 m height could not be used in most cases due to the difference in the house structures. Therefore, outdoor samples were sometimes collected at a height below or above 1.5 m.

### 2.3. Data Analysis

#### 2.3.1. Indoor and Outdoor PM_2.5_ Mass Concentration

Gravimetric weighing was undertaken to obtain the post-mass using the Sartorius electronic microbalance. Each filter was weighed three times before and three times after sampling and the average mass was recorded. The final mass was obtained by subtracting the initial mass from the post-mass of the filter, using Equation (1).

(1) $$M=\left(M_{f}-M_{i}\right)+\left(B_{f}-B_{i}\right)$$

where (*M*) is the final corrected mass (μg), (*M_f_*) is the post mass of the field filter after sampling, (*M_i_*) is the pre-mass of the field filter before sampling, (*B_f_*) is the post mass of the blank filter, (*B_i_*) is the pre mass of the blank filter. The volume of sampled air was obtained using Equation (2).

(2) $$V=\frac{fl\times t}{1000}$$

where (*V*) is the volume, (*fl*) is the flow rate at which the pump was sampling in L/min, (t) is the sampling duration in minutes. To convert the unit of volume from L/min to cubic metres (m^3^), the product of the flow rate and time was divided by 1000. The concentration of indoor and outdoor PM_2.5_ was calculated using Equation (3).

(3) $$C=\frac{M}{V}\;$$

where (*C*) is the concentration (μg/m^3^), (*M*) is the final corrected mass obtained using Equation (1) and (*V*) is the volume of sampled air in m^3^ obtained using Equation (2).

#### 2.3.2. Indoor–Outdoor Ratio

Indoor–outdoor ratios (I/O) were calculated to determine the difference between indoor and outdoor PM_2.5_ mass concentration and to determine whether there is a contribution of outdoor PM_2.5_ to the indoor environment. An I/O ratio of one indicates unity between the PM_2.5_ in the indoor and outdoor environment. An I/O ratio of less than one indicates a contribution of outdoor PM to the indoor environment. A ratio greater than one indicates a significant indoor source that is contributing to indoor air quality. The I/O ratio was obtained using Equation (4).

(4) $$C_{i}=\frac{C_{in}}{C_{out}}$$

where *C_i_* is the indoor-outdoor ratio, *C_in_* is the indoor PM_2.5_ mass concentration, and *C_out_* is the outdoor PM_2.5_ mass concentration.

#### 2.3.3. Statistical Analysis

Microsoft Excel 2019 version (Redmond, Washington, DC, USA) was used for data analysis and to compare the indoor and outdoor PM_2.5_ mass concentrations. An F-test was used to check the normality of the data and the type of t-test to employ. Based on the outcome of the F-test, Student’s t-test was used to test for a statistically significant difference between the means of indoor and outdoor PM_2.5_ mass concentrations. The Student’s t-test was performed at a 95% confidence level and a *p*-value of less than 0.05 indicated a statistically significant difference [49]. Microsoft Excel was also used to perform Pearson’s correlation coefficient to determine the strength of the relationship between indoor and outdoor PM_2.5_ mass concentrations. Regression was also performed to determine the contribution of outdoor PM_2.5_ on indoor PM_2.5_ mass concentrations. Furthermore, the indoor and outdoor PM_2.5_ mass concentration data was transferred to the IBM version 27 of the Statistical Package for Social Sciences software (Chicago, IL, USA), and a Tukey–Kramer post hoc test was performed to obtain an adjusted *p*-value.

#### 2.3.4. Scanning Electron Microscopy (SEM)

The morphology and chemical composition of the particles were studied using a Tescan Vega3 SEM (Tescan, Brno, Czech Republic) coupled with X-max 50 mm^2^ energy-dispersive spectroscopy (EDS) (Oxford Instruments, Abingdon, Oxfordshire, UK). SEM was used to image the surface of the particles and the EDS was used to semi-quantitatively analyse the chemical composition. Approximately 1 cm^2^ was cut from the centre of each selected PCTE filter containing the sampled PM, using a pair of scissors [50,51]. The centre of the analysed filters was assumed to be a representative of the entire deposited PM_2.5_ [52]. The filter pieces were mounted onto an aluminium stub, using a double-sided adherent carbon conductive tape [53]. The PCTE filters are non-conductive; therefore, the samples were sputter-coated with a thin layer of carbon (<10 nm) using an Agar Turbo Carbon coater (Agar Scientific, Stansted, UK). After coating, the stubs containing the carbon-coated samples were loaded into an SEM-EDS vacuum chamber for analysis.

The particle morphology was analysed using the back-scattered electron detector at a 20 kV accelerating voltage, an electron beam intensity of ~3 nA, at a working distance of 15 mm from the detector [54]. Similar to Kutchko and Kim [55], the elemental composition was analysed in a spot mode where the beam was localised on a single area that was manually chosen within the viewed sample. The different peaks were identified and the Oxford software Aztec (version 3.3 SPI) was used to obtain the peak intensities. The size of the particles was determined using version 1.46r of ImageJ (U.S. National Institute of Health, Bethesda, MD, USA), an open-source software package. The representative indoor and outdoor SEM images were loaded on the ImageJ software that counted the particle sizes automatically. Similar to Makonese et al. [56], a threshold was applied to the SEM images before analysis to obtain the best results. Thresholding is an automated process in which the image is converted into black and white, such that the black pixels represent particles and the white pixels are not particles [57]. The ImageJ software has been previously used in several studies to determine the number and size distribution of particles [58,59].

#### 2.3.5. Inductively Coupled Mass Plasma Spectrometry (ICP-MS)

The elemental composition of the particles was analysed using ICP-MS at the University of Johannesburg, Auckland Park campus, Spectrum laboratory. The sample filters were folded and placed inside pre-cleaned microwave digestion vessels; 9 mL ultrapure (Merck) nitric acid (HNO_3_) and 1 mL ultrapure (Merck) hydrogen peroxide (H_2_O_2_) was added to each vessel [60]. A reagent blank was included with the batch as a control. The vessels were closed and placed in a Mars 6 microwave (Mars CEM, Matthews NC, USA). The samples were transferred to a 50 mL volumetric flask of ultrapure water, 18.2 MΩ/cm resistivity using the Milli-Q system (Merck Millipore, Bedford, MA, USA). Calibration standards of 0, 0.1, 0.5, 1.0, 5.0, and 10 μg/L were prepared from 100 mg/L National Institute of Standards Technology traceable stock standards. The samples were then filtered using a 0.45 μm syringe filter and diluted 10 times (1 mL diluted to 10 mL). After digestion, the samples were analysed using a Perkin Elmer NexION 300 ICP-MS (Perkin Elmer, Waltham, MA, USA). The elements analysed using the ICP-MS included Mn, magnesium (Mg), silicon (Si), lead (Pb), vanadium (V), cadmium (Cd), sodium (Na), iron (Fe), cobalt (Co), nickel (Ni), copper (Cu), chromium (Cr), and zinc (Zn). Similar to Ari et al. [61], indoor and outdoor concentrations of the elements distributed on sampled PM_2.5_ were calculated using Equation (5).

(5) $$C_{e}=\frac{M_{e}-field\;blank\;filter}{V}$$

where (*C_e_*) is the concentration of the elements in micrograms (µg), (*M_e_*) is the mass of the elements from the ICP-MS in (µg), field blank filter is the mass of the blank filter, and (*V*) is the volume in cubic metre (m^3^).

### 2.4. Quality Control

Standard pendulums, weighing 100 and 200 g, were weighed on the microbalance before and after sampling to calibrate the scale and to validate the results. All filters were prepared in a dust-free laboratory; forceps were used to load and unload filters while wearing dust-free surgical gloves to avoid cross-contamination. A blank filter was prepared for each sampling period and transported to the laboratory with the field filters. During sampling, the blank filter was placed next to the field filters and used to account for moisture loss due to meteorological conditions, particularly during transportation. The flow rate of the pump was checked before and after sampling using a rotameter. The flow rate was verified using a bubble flow metre (Sensidyne, St Petersburg, FL, USA) and the fluctuation was within 5% deviation. Blank filters were also analysed using SEM-EDS and ICP-MS to obtain the background and to ensure that the filters were not contaminated.

## 3. Results

### 3.1. Indoor and Outdoor PM_2.5_ Mass Concentration

Table 1 presents the indoor and outdoor PM_2.5_ mass concentration together with the I/O ratio results. Mean indoor concentration ranged from 7.78 to 12.93 µg/m^3^ whereas the outdoor concentration ranged from 23.79–26.23 µg/m^3^. When comparing the overall combination of the indoor and outdoor PM_2.5_ concentrations for the three residential areas, the mean indoor and outdoor PM_2.5_ mass concentrations were 10.99 and 24.95 µg/m^3^, respectively. Furthermore, the outdoor PM_2.5_ mass concentration was 2.27-fold higher than the indoor concentration. From Table 1, it can also be observed that the outdoor PM_2.5_ concentrations were higher than the indoor concentrations across the three residential areas. Outdoor PM_2.5_ concentrations in Old Sicelo, New Sicelo, and Noldick were 1.94-, 1.92-, and 3.37-fold higher than the indoor concentrations, respectively. These findings suggest that the indoor environments were influenced by PM_2.5_ from the outdoor environment. A statistically significant difference (*p* < 0.05) was found, implying that there was a significant difference between the means of the indoor and outdoor PM_2.5_ mean concentrations across the three residential areas. The highest mean I/O ratio was recorded at New Sicelo whereas the lowest was recorded at Noldick. Mean I/O ratios across the three residential areas were less than one, indicating that indoor PM_2.5_ mass concentrations were influenced by PM_2.5_ from the outdoor environment.

Table 2 shows the Pearson’s correlation coefficients and regression results between indoor and outdoor PM_2.5_ mass concentrations. It can be observed that there was a strong positive relationship between indoor and outdoor PM_2.5_ mass concentrations across the three residential areas. Approximately 94% of the indoor PM_2.5_ concentrations in Old Sicelo could be explained by the outdoor PM_2.5_, whereas 80 and 76% of indoor PM_2.5_ in New Sicelo and Noldick, respectively, could be explained by outdoor PM_2.5_.

### 3.2. Morphology of Indoor and Outdoor PM_2.5_

**Figure 2:**
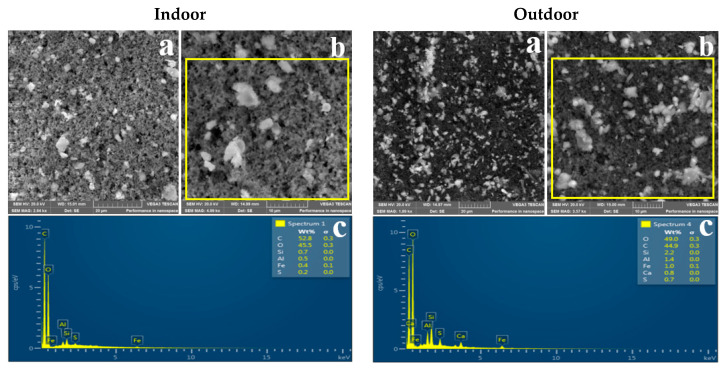
SEM images of indoor and outdoor PM_2.5_ sampled at Old Sicelo: (**a**) image captured at 20 µm; (**b**) image captured at 10 µm; (**c**) EDS spectrum of the portion highlighted in yellow.

**Figure 3:**
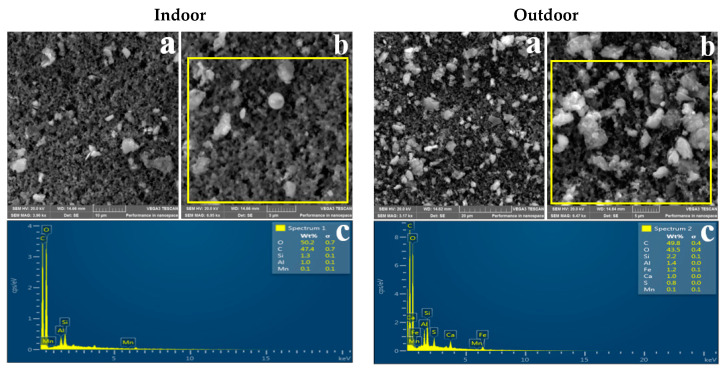
SEM images of indoor and outdoor PM_2.5_ sampled at New Sicelo: (**a**) indoor image captured at 10 µm; (**b**) indoor captured at 5 µm; (**a**) outdoor image captured at 20 µm; (**b**) image captured at 5 µm; (**c**) EDS spectrum of the portion highlighted in yellow.

**Figure 4:**
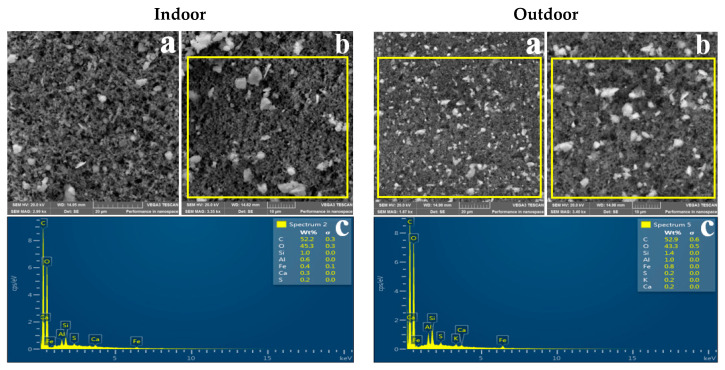
SEM images of indoor and outdoor PM_2.5_ sampled at Noldick: (**a**) image captured at 20 µm; (**b**) image captured at 10 µm; (**c**) EDS spectrum of the portion highlighted in yellow.

Representative SEM images showing the shape of indoor and outdoor PM_2.5_ sampled in the three residential areas are shown in Figure 2, Figure 3 and Figure 4. It can be observed that indoor and outdoor PM_2.5_ had different sizes and shapes. Indoor PM_2.5_ in all three areas consisted of irregular and spherical particles, whereas outdoor PM_2.5_ consisted of agglomerated and irregular-shaped particles. PM_2.5_ from the indoor and outdoor environments had the same or similar morphologies, suggesting that it was from the same source.

Table 3 shows particle diameter results for the indoor and outdoor PM_2.5_ sampled in the three residential areas. The mean size of the indoor particles ranged between 0.38 and 0.49 µm, whereas the mean size of outdoor particles ranged between 0.3 and 0.37 µm. It can be observed that both indoor and outdoor PM_2.5_ in the three residential areas were in the accumulation mode (0.1–1 µm).

### 3.3. Elemental Composition of Indoor and Outdoor PM_2.5_

Table 4 shows the concentrations of elements in the air in indoor and outdoor environments obtained using ICP-MS. It can be observed that outdoor concentrations of elements were higher than indoor concentrations across the three residential areas, also suggesting that the indoor environment was impacted by PM_2.5_ from the outdoor environment. Silicon, Mn, and iron were the highest in both indoor and outdoor environments. Potassium and cadmium were not detected in either indoor and outdoor samples across the three residential areas. The limits of quantification for K and Cd were <10.67 and <0.033, respectively.

## 4. Discussion

### 4.1. Indoor and Outdoor PM_2.5_ Mass Concentration

New Sicelo had the highest average indoor PM_2.5_ mass concentration (12.9 ± 3.3 µg/m^3^), followed by Old Sicelo (12.3 ± 4.3 µg/m^3^) and Noldick (7.78 ± 6.1 µg/m^3^) (Table 1). The high indoor PM_2.5_ mass concentrations at New and Old Sicelo suggest that the sampled households had many or larger openings, enabling the PM_2.5_ to infiltrate at a faster rate, whereas the lower indoor PM_2.5_ mass concentrations at Noldick suggest that outdoor PM_2.5_ infiltrated at a lower rate. These results were expected because most of the sampled households in Noldick are made of brick and cement and have ceilings, whereas those in Old and New Sicelo are predominantly shacks made of corrugated iron and cardboard. The infiltration of outdoor PM into the indoor environment depends on the characteristics of the structure and integrity of the building envelope [62,63]. Studies by Lv et al. [64], Martins et al. [65], and Zhang et al. [62] found that structures with openings tended to have a higher penetration of PM. Therefore, the different housing structures in New Sicelo, Old Sicelo, and Noldick may explain the variation in indoor PM_2.5_ mass concentrations. However, it is also possible that the PM_2.5_ did not only enter through openings but also through foot tracking, whereas some could have been suspended or resuspended during walking and cleaning activities [66,67,68,69].

Noldick had the highest average outdoor PM_2.5_ mass concentration (26.23 ± 5 µg/m^3^), followed by New Sicelo (24.89 ± 9 µg/m^3^), and Old Sicelo (18.7 ± 8.9 µg/m^3^) (Table 1). However, the 24 h mean outdoor PM_2.5_ mass concentrations across the three residential areas were within the World Health Organization ambient air quality guidelines and the South African national air quality standard of 25 µg/m^3^. Although Noldick is furthest from the FeMn smelter, it had the highest outdoor PM_2.5_ mass concentration relative to New Sicelo, which is nearest. This finding is an indication that the FeMn smelter is not the only contributing source of outdoor PM_2.5_; the coal-fired starch factory, cement factory, and mobile sources are also potential sources. Moreover, Noldick is closest to the R59 highway, R551 and M61 main roads, the coal-fired starch factory, and the cement factory.

Our indoor and outdoor PM_2.5_ mass concentration results are similar to those of previous studies [70,71,72], which also found that the outdoor mass concentration was greater than the indoor concentration. Studies by Martuzevicius et al. [73] and Abdel-Salam [74] found a significant correlation between the distance from major traffic roads and outdoor PM_2.5_ mass concentrations in residential areas. The difference in outdoor PM_2.5_ mass concentrations across the three residential areas could be due to variation in the source, source strength, distance from the source, and different meteorological conditions [7,8,75,76].

### 4.2. Relationship between Indoor and Outdoor PM_2.5_ Mass Concentrations

As shown in Table 1, the mean I/O ratio at New and Old Sicelo showed less variation; however, the I/O ratios of the two residential areas varied when compared to Noldick. Nonetheless, the I/O ratios across the three residential areas were below one, implying that the indoor PM_2.5_ mass concentrations were influenced by PM_2.5_ from the outdoor environment. The correlation coefficient results (Table 2) showed a strong positive relationship between indoor and outdoor PM_2.5_ mass concentrations across the three residential areas. Correlation coefficients for Old Sicelo, New Sicelo, and Noldick were r = 0.97, *p* = 0.03; r = 0.90, *p* = 0.01; and r = 0.88, *p* = 0.01, respectively. The R-square values of Old Sicelo, New Sicelo, and Noldick were 0.94, 0.80, and 0.76, respectively.

The relationship between indoor and outdoor PM_2.5_ mass concentrations in Old Sicelo can be described using the equation y = −3.96x + 0.68, which indicates that for every unit mass increase in outdoor PM_2.5_, the indoor mass concentration increases by 0.68 µg/m^3^. At New Sicelo, the relationship between indoor and outdoor PM_2.5_ concentrations can be described by the equation y = 4.81x + 0.33, which indicates that for every unit mass increase in outdoor PM_2.5_, indoor mass concentration increases by 0.33 µg/m^3^. The indoor and outdoor PM_2.5_ mass concentration relationships at Noldick can be represented using the equation y = −19.87x + 1.05, which indicates that for every unit mass increase in outdoor PM_2.5_, indoor mass concentration increases by 1.05 µg/m^3^ 0.33 µg/m^3^.

The positive correlation between indoor and outdoor PM_2.5_ concentrations, R-square values close to one, and the corresponding I/O ratios of less than one, confirm that indoor concentrations were influenced by PM_2.5_ from the outdoor environment. Similar results were reported by Massey et al. [77] and Bozlaker et al. [72], who also found a strong and positive relationship between indoor and outdoor PM_2.5_ mass concentrations. The results are expected because it has been reported that approximately 35–70% of indoor PM_2.5_ comes from the outdoor environment [64,78]. Our indoor and outdoor PM_2.5_ mass concentration findings complement those of Hasheminassab et al. [13], Gao et al. [79], and Zhao et al. [80], who found that outdoor PM_2.5_ mass concentration was significantly higher than the indoor concentration.

### 4.3. Morphology

SEM-EDS was used to determine the physicochemical properties of indoor and outdoor PM_2.5_. Figure 2, Figure 3 and Figure 4 show representative SEM-EDS images of indoor and outdoor PM_2.5_ for the three residential areas. As previously mentioned, indoor PM_2.5_ across the three residential areas consisted of irregular and spherical particles, whereas the outdoor PM_2.5_ was dominated by compact agglomerated irregular particles. Mn particles tend to form aggregates of primary particles that are fused and agglomerates of string-like clusters of primary particles that adhere due to electrostatic forces [81,82]. According to Gjønnes et al. [83], agglomerated particles indicate that the particles were of submicron size and agglomerated to form larger particles. Spherical particles commonly originate from natural processes such as pollen, or anthropogenic sources such as high-temperature combustion processes [4,50,84]. Therefore, it can be concluded that the indoor spherical particles are from a high-combustion source(s) such as the nearby FeMn smelter.

Table 3 shows the indoor and outdoor PM_2.5_ particle diameter results. It can be observed that indoor particles were larger than the outdoor particles; however, both indoor and outdoor particles were of a submicron size. The mean particle diameter for indoor PM_2.5_ was 0.49 µm for Old Sicelo, 0.38 µm for New Sicelo, and 0.40 µm for Noldick. The mean outdoor particle diameters at Old Sicelo, New Sicelo, and Noldick were 0.37, 0.36, 0.30 µm, respectively. Our SEM results are similar to those of Gjønnes et al. who used SEM-EDS to investigate the physicochemical properties of particulate matter during various processes in a ferro and silicomanganese smelter in Norway. The authors reported spherical, irregular, and agglomerated submicron Mn oxide particles. Our results also complement those of Ervik et al. [85], who conducted a study in a ferro and silicomanganese smelter in Norway using SEM-EDS and found that the FeMn smelter was dominated by compact agglomerated and individual spherical oxidic Mn particles. The authors also reported elements such as Mn, sulphur, and silicon, which were also found in this study. In another study conducted near a FeMn alloy plant in Cantabria, Spain, Hernández-Pellón et al. [7] found irregular Mn- and Fe-enriched particles.

### 4.4. Elemental Composition of Indoor and Outdoor PM_2.5_

The EDS results revealed that indoor and outdoor PM_2.5_ across the three residential areas was enriched with elements such as aluminium, iron, and sulphur. Potassium and cadmium were not detected in either indoor and outdoor samples across the three residential areas, for which the limit of detection for the EDS was <0.1 weight percent (Wt%). EDS is semi-quantitative; therefore, limited conclusions can be made from it. Hence, in this study, ICP-MS was used to overcome this limitation. A summary of the elemental composition of indoor and outdoor PM_2.5_ obtained using ICP-MS is presented in Table 4. All elements selected for ICP-MS analysis were detected, with the exception of potassium and cadmium, which were below the limit of quantification. EDS analysis (4c) shows that potassium was detected (0.20 Wt%) in the outdoor PM_2.5_; however, it was below the limit of quantification of the ICP-MS, even though ICP-MS is more sensitive relative to EDS. This is because the filters that were analysed using SEM-EDS were not the same filters analysed using ICP-MS. The potassium finding is an indication that there were biomass burning activities at Noldick because potassium is associated with the combustion of solid fuels such as wood and coal. Therefore, the potassium concentration at Noldick can be attributed to the nearby coal-fired starch factory or residential solid fuel burning.

From Table 4, it can be observed that the indoor and outdoor PM_2.5_ across three residential areas was dominated by elements in the decreasing order of Si > Fe > Mn. However, outdoor concentrations of elements in the air were higher than the indoor concentrations, further supporting the indoor and outdoor PM_2.5_ mass concentrations reported in Table 1. The elements reported in Table 4 have been reported in residential areas near FeMn smelters and high traffic density [8,86]. Noldick had the highest indoor Mn concentrations (1.7 µg/m^3^), followed by Old Sicelo (1.2 µg/m^3^), whereas New Sicelo had the lowest concentration (1.03 µg/m^3^). Noldick, which is 1.5 km from the FeMn smelter, also had the highest outdoor Mn concentration (6.7 µg/m^3^), followed by New Sicelo (5.6 µg/m^3^), which is 1.4 km away. Old Sicelo, which is located 3.5 km from the FeMn smelter, reported the least airborne Mn concentration (3.9 µg/m^3^). Indoor Fe concentrations at Old Sicelo, New Sicelo, and Noldick were 3.87, 7.08, and 4.85 µg/m^3^, respectively, whereas outdoor Fe concentrations were 10.53, 12.06, and 6.17 µg/m^3^, respectively.

The indoor and outdoor Fe and Mn concentrations can be attributed to the FeMn smelter and vehicular emissions [7,87]. Studies have found higher Mn concentrations in residential areas near FeMn smelters, particularly in residential areas downwind. A study conducted in residential areas near a FeMn metallurgy plant in Boulogne-Sur-Mer, France, found high Fe and Mn concentrations in a residential area within 200 m [88]. In another study conducted in Cantabria, Spain, Expósito et al. [76] found higher concentrations of airborne Mn concentrations in residential areas within 1.5 km of the FeMn smelter. The airborne Fe concentrations can also be attributed to the corrugated Fe used for roofing and constructing shacks.

After the ban of Pb in the petroleum industry, methylcyclopentadienyl manganese tricarbonyl, which is an organic derivative of Mn, has been used significantly as an additive to improve the octane level and anti-knock characteristic of petrol [89,90]. Mn air concentrations in urban areas are influenced by traffic resuspension, abrasion of brake pads, and the combustion of methylcyclopentadienyl manganese tricarbonyl [22]. Sanderson et al. [91] reported that engine blocks can contribute significantly to the ambient Fe concentration. A study conducted in three residential areas near an industrial site in Durban, South Africa, found high Mn concentrations, which were attributed to metal works industries and high traffic density in one of the residential areas studied [92]. Silicon is a naturally occurring element that is abundant in the Earth’s crust and is also a marker of coal-burning [93,94]. Therefore, the high presence of Si content in indoor and outdoor PM_2.5_ can be attributed to the coal-fired starch factory, the FeMn smelter, and the cement factory.

The cement factory, motor vehicular emissions, the coal-fired starch factory and the FeMn smelter were identified as potential contributing sources of indoor and outdoor PM_2.5_ in three residential areas in Meyerton. The physicochemical properties of indoor and outdoor PM_2.5_ suggest that the nearby FeMn smelter is the main potential emission source. Our findings suggest that there is a need for integrated town planning and development strategies whereby FeMn smelters should not be developed near residential areas. Similarly, residential areas should be developed near FeMn smelters, particularly downwind. Our findings can be used to strengthen epidemiological data and for human health risk assessment. Although the current study was focused and confined to the characterisation of indoor and outdoor PM_2.5_, the findings indicate that there is potential for exposure to PM_2.5_ enriched with elements both in indoor and outdoor environments. Therefore, the following are recommended to protect the health of residents in the three sampled residential areas in Meyerton: tree plantation is recommended to trap atmospheric PM, and planting of vegetation and paving of dusty areas should be implemented to prevent the resuspension of settled PM by wind or human activities. The planting of trees should also be undertaken alongside the busy roads because motor vehicular emissions were identified as the major potential source of Mn- and Fe-enriched PM_2.5_.

Interventional studies are important given the health outcomes associated with exposure to PM_2.5_ enriched with elements, particularly Mn-bearing PM_2.5_. For example, the interventional studies can investigate the type of interventions needed and where they can be placed to effectively reduce exposure to PM_2.5_ Mn-bearing particles, both indoor and outdoor. Exposure and human health risk assessment studies are also recommended in the Meyerton area. Where possible, the measurements should be taken at the breathing zone of the receptors, e.g., [25,95,96], to reduce the uncertainty. Future studies can investigate the environmental and human health impacts of the FeMn smelter and motor vehicular emissions beyond the three residential areas studied. Furthermore, future studies can investigate the possibility of the PM_2.5_ changing from the air compartment to water and soil. Time–activity pattern studies are also recommended to quantify how much time the residents spend indoors, outdoors, and outside the boundaries of their microenvironment. Time-activity pattern studies are necessary because the development of adverse health outcomes depends on factors such as duration and frequency of exposure. Therefore, time–activity patterns can be used to collect data that will be useful for exposure assessment and modelling the intake and uptake of PM_2.5_.

### 4.5. Strengths and Limitations

This is the first study to investigate the physicochemical properties of indoor and outdoor PM_2.5_ in a residential area near a FeMn smelter in South Africa. The study did not use data obtained from stationery monitors located farther from the receptors; rather, PM_2.5_ samples were collected at the level of the receptors. PM_2.5_ samples were also collected in indoor environments, which are neglected in numerous studies, even though people spend 80–90% of their time indoors. The study also identified the major potential emitting sources of indoor and outdoor PM_2.5_-bound elements in the three residential areas.

We only analysed a small portion of the filter (~1 cm^2^) cut from the centre of the filter. We acknowledge that some of the sampled PM_2.5_ could have deposited on the edge of the filters. Due to limited funds, only two indoor and outdoor filters per residential area were analysed using ICP-MS. The study did not use a weather monitoring station to obtain meteorological data, which are important for the transportation and dispersion of atmospheric PM. Moreover, there was no monitoring data for Meyerton on the South African Air Quality Information System during the study. Due to this limitation, the PM_2.5_ concentrations were not correlated with meteorological data, such as wind speed, wind direction, humidity, and temperature. Time–activity patterns were also not included in this study. The time–activity patterns would have assisted in understating the contribution of indoor sources to the indoor PM_2.5_ mass concentration.

The data were collected during the spring season, and the PM_2.5_ concentrations are unlikely to remain consistent throughout the different seasons. Therefore, future studies should be conducted over a longer period to address seasonal variations. The air exchange rates in the sampled households were not measured, and may have helped determine the infiltration and deposition rate of outdoor PM_2.5_ into the indoor environments. Information about the production rate of the smelter was also unobtainable. Although the sample size was small, there is no reason to believe that the measurements were not representative of the three areas. A study using a larger sample size could, however, be conducted to confirm the findings.

## 5. Conclusions

In this study, indoor and outdoor PM_2.5_ samples were collected concurrently in three residential areas near a FeMn smelter. We characterised indoor and outdoor PM_2.5_, determined the relationship between indoor and outdoor PM_2.5_ mass concentrations, and apportioned the potential source(s). For the first time in the context of South Africa, the physicochemical properties of indoor and outdoor PM_2.5_ in a residential area near a FeMn smelter were investigated. Therefore, the findings can be used as baseline information because such data is limited in South Africa. Indoor PM_2.5_ mass concentrations were less than the outdoor concentrations in all three residential areas, and the mean outdoor mass concentration was 2.27-fold higher than the indoor concentration. Pearson’s correlation coefficients showed a strong positive relationship between indoor and outdoor PM_2.5_ mass concentrations in the three residential areas. The mean I/O ratios of less than one and a positive relationship between indoor and outdoor PM_2.5_ supported the hypothesis that indoor PM_2.5_ mass concentrations were influenced by PM_2.5_ from the outdoor environment. EDS and ICP-MS analysis showed the presence of elements commonly associated with FeMn smelter emissions. The concentrations of elements were higher in outdoor environments than in indoor environments across the three residential areas. The spherical, irregular, and agglomerated submicron particles, and their elemental composition, are associated with high-combustion sources such as FeMn smelter and motor vehicles. Therefore, the coal-fired starch factory, the FeMn smelter, motor vehicles, and the FeMn smelter are potential sources of indoor and outdoor PM_2.5_ of different physicochemical properties in the three residential areas in Meyerton.

## Figures and Tables

**Table 1:** Descriptive statistics of indoor and outdoor PM_2.5_ mass concentrations (µg/m^3^) and indoor–outdoor ratios in the three residential areas.

Residential Area	Pairings	Min	Max	Mean	SD	%Difference	I/O	CI (95%)	
								Q1	Q3
Old Sicelo	Indoor	5.9475	18.840	12.2516	4.3295			8.2864	16.1538
	vs.					48.5095	0.5040		
	Outdoor	15.2314	33.9638	23.7939	6.1595			18.2963	28.8151
New Sicelo	Indoor	8.3359	19.1895	12.9288	3.290			10.4902	15.9271
	vs.					48.0221	0.5441		
	Outdoor	11.680	40.4385	24.8737	9.0046			18.4296	31.4255
Noldick	Indoor	2.8828	16.9837	7.7841	6.0794			3.6197	15.9553
	vs.					70.3243	0.2732		
	Outdoor	19.470	35.0946	26.2305	5.0204			21.8400	30.2382

Min: minimum; Max; maximum; SD: standard deviation; I/O: indoor–outdoor ratio; CI: confidence interval; Q1: lower quartile; Q3: upper quartile.

**Table 2:** Correlation and regression between indoor and outdoor PM_2.5_ mass concentrations for the three residential areas.

Residential Area	r-Value	Intercept	Slope	R-Square	*p*-Value	Adjusted *p*-Value
Old Sicelo	0.9688	−3.9517	0.6810	0.9386	0.0307	0.0038
New Sicelo	0.8932	4.8116	0.3263	0.7978	0.0136	0.0025
Noldick	0.8703	−19.8615	1.0539	0.7575	0.0077	0.0010

**Table 3:** Indoor and outdoor particle diameter (µm) measurements in the three residential areas.

Parameter	Old Sicelo		New Sicelo		Noldick	
	Indoor	Outdoor	Indoor	Outdoor	Indoor	Outdoor
Minimum	0.09	0.11	0.10	0.10	0.10	0.14
Mean	0.49	0.37	0.38	0.36	0.40	0.30
Maximum	1.06	0.67	0.66	0.70	0.70	0.48

**Table 4:** Indoor and outdoor elemental concentrations (µg/m^3^) in the air across the three residential areas.

Elements	Old Sicelo		New Sicelo		Noldick	
	Indoor	Outdoor	Indoor	Outdoor	Indoor	Outdoor
Si	4.7417	11.3543	6.2718	15.3786	5.3376	7.5356
Fe	3.8665	10.5339	7.0881	12.0622	4.8544	6.1707
Mg	1.5450	1.9122	0.6943	1.1227	0.8178	0.9574
Mn	1.2045	3.9739	1.0346	5.5495	1.6712	6.5604
Na	0.9193	2.9715	1.1837	2.0935	0.8950	0.7115
Zn	0.1422	1.7705	0.3307	0.7567	0.3446	0.5178
Cr	0.0586	0.3284	0.0540	0.0631	0.0610	0.5136
Pb	0.0258	0.2958	0.0656	0.1037	0.0494	0.5098
Cu	0.0241	0.3270	0.0317	0.2742	0.0866	0.2819
Ni	0.0123	0.0882	0.0074	0.0510	0.0222	0.0800
Co	0.0046	0.0667	0.0073	0.0091	0.0074	0.0080
V	0.0024	0.2045	0.0225	0.0268	0.0225	0.0544
K	<LoQ	<LoQ	<LoQ	<LoQ	<LoQ	<LoQ
Cd	<LoQ	<LoQ	<LoQ	<LoQ	<LoQ	<LoQ

<LoQ: below limit of quantification.

## Data Availability

The data presented in this study are available on reasonable request from the corresponding author.
